# The Antiviral Activity of the Cellular Glycoprotein LGALS3BP/90K Is Species Specific

**DOI:** 10.1128/JVI.00226-18

**Published:** 2018-06-29

**Authors:** Veronika Lodermeyer, George Ssebyatika, Vânia Passos, Aparna Ponnurangam, Angelina Malassa, Ellen Ewald, Christina M. Stürzel, Frank Kirchhoff, Margalida Rotger, Christine S. Falk, Amalio Telenti, Thomas Krey, Christine Goffinet

**Affiliations:** a Institute of Molecular Virology, Ulm University Medical Center, Ulm, Germany; b Institute of Virology, Hannover Medical School, Hannover, Germany; c Institute for Experimental Virology, Twincore Centre for Experimental and Clinical Infection Research, Hannover, Germany; d Institute of Microbiology, University Hospital Center, University of Lausanne, Lausanne, Switzerland; e Institute of Transplant Immunology, Integrated Research and Treatment Center Transplantation, IFB.Tx, Hannover Medical School, Hannover, Germany; f Human Longevity Inc., San Diego, California, USA; g German Center for Infection Research (DZIF), Hannover, Germany; Emory University

**Keywords:** antiviral, human immunodeficiency virus, interferons, restriction factor, virus infectivity

## Abstract

Cellular antiviral proteins interfere with distinct steps of replication cycles of viruses. The galectin 3 binding protein (LGALS3BP, also known as 90K) was previously shown to lower the infectivity of nascent human immunodeficiency virus type 1 (HIV-1) virions when expressed in virus-producing cells. This antiviral effect was accompanied by impaired gp160Env processing and reduced viral incorporation of mature Env glycoproteins. Here, we examined the ability of 90K orthologs from primate species to reduce the particle infectivity of distinct lentiviruses. We show that 90K's ability to diminish the infectivity of lentiviral particles is conserved within primate species, with the notable exception of 90K from rhesus macaque. Comparison of active and inactive 90K orthologs and variants uncovered the fact that inhibition of processing of the HIV-1 Env precursor and reduction of cell surface expression of HIV-1 Env gp120 are required, but not sufficient, for 90K-mediated antiviral activity. Rather, 90K-mediated reduction of virion-associated gp120 coincided with antiviral activity, suggesting that 90K impairs the incorporation of HIV-1 Env into budding virions. We show that a single “humanizing” amino acid exchange in the BTB (broad-complex, tramtrack, and bric-à-brac)/POZ (poxvirus and zinc finger) domain is sufficient to fully rescue the antiviral activity of a shortened version of rhesus macaque 90K, but not that of the full-length protein. Comparison of the X-ray structures of the BTB/POZ domains of 90K from rhesus macaques and humans point toward a slightly larger hydrophobic patch at the surface of the rhesus macaque BTB domain that may modulate a direct interaction with either a second 90K domain or a different protein.

**IMPORTANCE** The cellular 90K protein has been shown to diminish the infectivity of nascent HIV-1 particles. When produced in 90K-expressing cells, particles bear smaller amounts of the HIV-1 Env glycoprotein, which is essential for attaching to and entering new target cells in the subsequent infection round. However, whether the antiviral function of 90K is conserved across primates is unknown. Here, we found that 90K orthologs from most primate species, but, surprisingly, not from rhesus macaques, inhibit HIV-1. The introduction of a single amino acid exchange into a short version of the rhesus macaque 90K protein, consisting of the two intermediate domains of 90K, resulted in full restoration of antiviral activity. Structural elucidation of the respective domain suggests that the absence of antiviral activity in the rhesus macaque factor may be linked to a subtle change in protein-protein interaction.

## INTRODUCTION

Mammalian cells express an arsenal of genes which encode antiviral proteins. The expression of most, but not all, of them is upregulated by type I interferons. CNP (1), Tetherin (2, 3), and GBP5 (4), proteins of the IFITM family (5, 6), and SERINC5 (7, 8) block particle assembly, block particle release, or reduce HIV-1 particle infectivity, demonstrating that late steps of the lentiviral replication cycle can be targeted by host cell defenses. The cellular glycoprotein LGALS3BP (galectin 3 binding protein, also known as 90K) is a secreted antiviral protein that has been identified by us (9) and others (10) to interfere with late steps of human immunodeficiency virus type 1 (HIV-1) biogenesis, resulting in poor infectivity of released virions. 90K was originally identified as a tumor antigen, and its expression is also upregulated in the context of HIV-1 and hepatitis C virus (HCV) infections, reaching secreted protein levels in the microgram-per-milliliter range in body fluids (11–13). 90K is composed of a scavenger receptor cysteine rich-like domain of known structure (14), a BTB (broad-complex, tramtrack, and bric-à-brac)/POZ (poxvirus and zinc finger) domain, an IVR (intervening region) domain, and a C-terminal part without substantial homology to known protein domains (15). Upon completion of posttranslational modifications, the highly glycosylated 90K is secreted in an ERGIC-53-regulated manner (16) and associates with the extracellular matrices of various tissues, where it oligomerizes to ring-like and linear structures (15, 17, 18). Physiological functions of the extracellular protein may comprise immunomodulation and modulation of proliferation, motility, migration, and adhesion of cells via interaction with cell surface receptors (19). Specifically, 90K-mediated induction of cell signaling involves upregulation of cytokine production, including interleukin 2 (IL-2), IL-6, granulocyte-macrophage colony-stimulating factor (GM-CSF), and tumor necrosis factor alpha (TNF-α) (19–21), which may contribute to an antiviral state. We have shown previously that 90K expressed within HIV-1-producing cells mediates the antiviral activity against HIV-1, but not exogenously added secreted protein, suggesting an intracellular mode of antiviral action (9). Inhibition correlated with reduced viral incorporation of mature HIV-1 gp120 and gp41 glycoproteins and was accompanied by reduced HIV-1 gp160 Env precursor processing. Importantly, small interfering RNA (siRNA)-mediated reduction of 90K protein expression increased particle infectivity of released virions in TZM-bl cell lines by 10-fold and enhanced the spread of HIV-1 Ba-L in primary macrophages up to 5-fold, indicating that physiological levels of 90K expression contribute to HIV-1 restriction (9).

Investigating the ability of closely related orthologs of restriction factors to inhibit viral infections is a valuable approach to identifying domains that determine antiviral activity and to shedding light on the mode of antiviral action. Here, we address the species specificity of 90K orthologs to restrict lentiviruses, and we identify functional and structural determinants of the antiviral activity of 90K.

## RESULTS

### Species-specific antiviral activity of 90K proteins.

**FIG 1:**
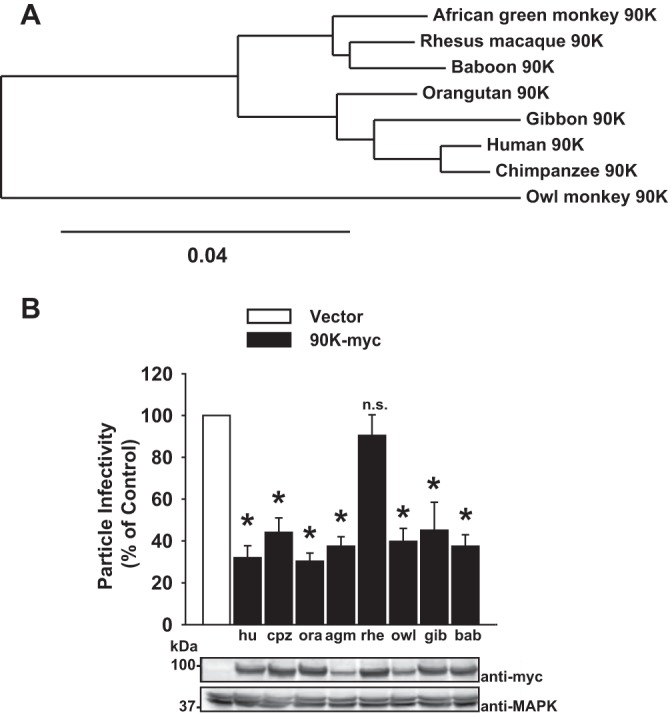
Species-specific antiviral activity of 90K proteins. (A) Phylogenetic tree of 90K proteins, generated with phylogeny.fr (40). (B) HEK293T cells were cotransfected with 1.3 μg HIV-1 proviral DNA and 1.3 μg of the indicated constructs. The abbreviations for the individual species are defined in Materials and Methods. At 48 h posttransfection, the infectivity of cell-free particles was calculated as infectivity per nanogram of p24 capsid antigen in the supernatant. Particle infectivity in the absence of 90K was set to 100%. Shown are mean values from 3 to 4 independent experiments ± SEM. Lysates of the producer cells were immunoblotted with anti-myc and anti-MAPK antibodies. Asterisks indicate statistical significance (*P* < 0.05); n.s., not significant.

The coding sequence of 90K is highly conserved within primates, with amino acid homologies ranging from 90 to 98% between individual nonhuman primate 90K proteins and human 90K (Fig. 1A). To explore the species specificity of 90K's antiviral activity, we analyzed the infectivity of HIV-1 particles generated in producer HEK293T cells expressing heterologous, C-terminally myc-tagged 90K proteins. Importantly, HEK293T cells lack detectable expression of endogenous 90K protein (9) and therefore represent a suitable system for heterologous expression studies. The infectivity of released particles, defined as infectivity per nanogram of HIV-1 p24 capsid in the culture supernatant, was diminished by 70% by human 90K-myc (Fig. 1B), confirming our previous data (9). All seven tested nonhuman 90K proteins, with the surprising exception of 90K from rhesus macaques, reduced particle infectivity to similar extents. Anti-myc immunoblotting confirmed that all proteins were expressed (Fig. 1B). These results point toward an evolutionarily conserved, but not universal, antiviral activity of 90K proteins among primate species that manifests itself as a decreased infectivity of secreted HIV-1 particles.

### 90K inhibits HIV-1 by impairing Env incorporation into particles.

**FIG 2:**
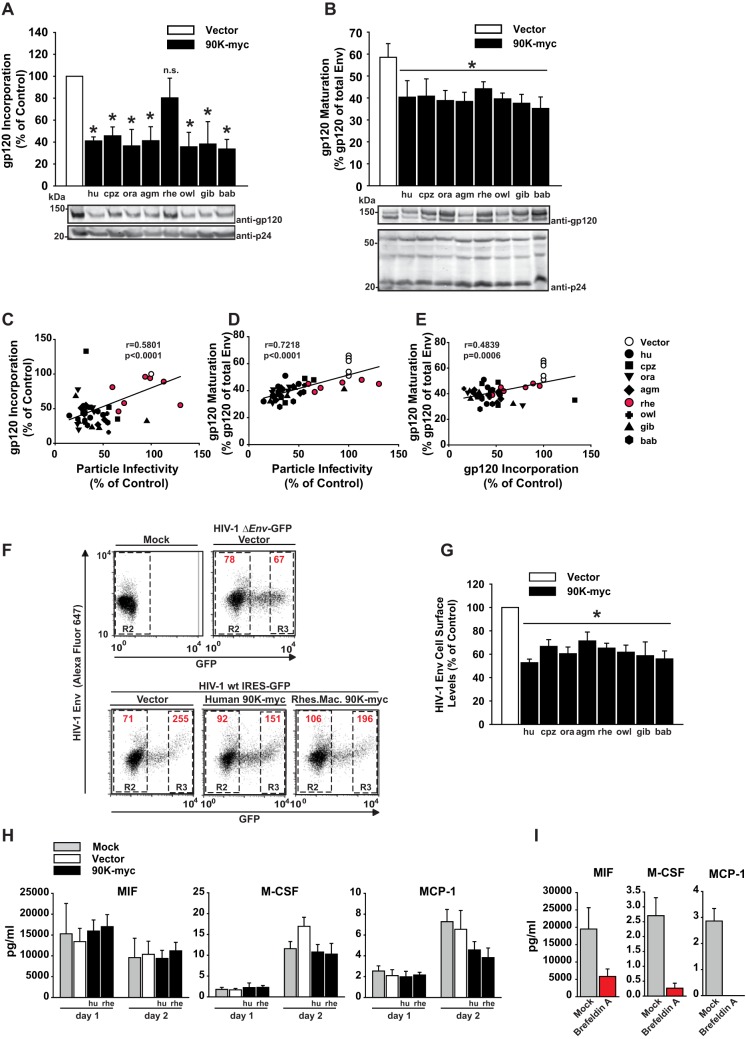
90K inhibits HIV-1 by impairing Env incorporation into particles. (A) Particles for which results are shown in Fig. 1B were sucrose cushion-purified and analyzed by immunoblotting for gp120 incorporation using anti-gp120 and anti-p24 antibodies. gp120 incorporation was calculated as the ratio of gp120 signal intensity to p24 signal intensity. Values obtained in the absence of 90K were set to 100%. Asterisks indicate statistical significance (*P* < 0.05); n.s., not significant. (B) Producer cell lysates were analyzed by immunoblotting for gp160 processing using anti-gp120 and for Gag expression using anti-p24. Relative gp160 processing was determined by expressing the level of mature gp120 as a percentage of the whole-Env (gp120 + gp160) signal. Band intensities were quantified by infrared-based imaging. The bar diagrams show mean values from 3 to 7 independent experiments ± SEM. (C) Correlative analysis of gp120 Env content per p24 CA and HIV-1 particle infectivity (infectivity per nanograms of p24) in the supernatants of cells expressing the indicated 90K-myc protein or the vector. (D) Correlative analysis of HIV-1 particle infectivity and levels of mature gp120 per whole-Env expression in producer cell lysates expressing the indicated 90K-myc protein or the vector. (E) Correlative analysis of levels of gp120 viral incorporation and levels of mature gp120 per whole-Env expression in producer cell lysates. In panels C to E, symbols represent the values for individual transfections. The Pearson correlation coefficient *r* and the corresponding *P* value were calculated using GraphPad Prism software. (F) HEK293T cells were mock transfected or cotransfected with the indicated HIV-1 proviral DNAs and constructs. At 48 h posttransfection, intact cells were immunostained for HIV-1 Env surface expression and analyzed by flow cytometry. In the dot plots, the HIV-1 Env levels are plotted against GFP. Numbers inside the boxes indicate the mean fluorescence intensity (MFI) of the HIV-1 Env signal in the respective gates. (G) The levels of cell-surface-exposed HIV-1 Env in each sample were quantified on GFP-positive cells in the R3 gate relative to the MFI of GFP-negative cells in the R2 gate, which serve as an internal reference. The relative cell surface expression levels of HIV-1 Env are depicted, with values for HIV-1 Env expression in vector-transfected cells set to 100%. Shown are mean values from six independent experiments ± SEM. (H) HEK293T cells were either mock treated or transfected with an empty vector or 90K-encoding plasmids. Supernatants were harvested at days 1 and 2 posttransfection and were subjected to Luminex-based multiplex assays according to the manufacturer's instructions. Secretion into supernatants was analyzed for MIF, M-CSF, and MCP-1 (CCL2); all concentrations are given in picograms per milliliter. (I) HEK 293T cells were either mock treated or brefeldin A treated for 6 h. Supernatants were then subjected to Luminex-based multiplex assays as outlined for panel H.

Human 90K-mediated reduction of particle infectivity correlates with decreased levels of incorporated gp120 glycoproteins and is accompanied by lowered Env cell surface expression and reduced cleavage efficiency of the glycoprotein precursor (9). Here, we investigated the protein composition of virus particles produced in the presence of primate 90K proteins and determined the cleavage efficiency of the Env polyprotein precursor within the respective HIV-1 producer cells by quantitative immunoblotting. Levels of virus-associated mature gp120 were reduced 54 to 66% by human 90K and the six active nonhuman primate variants but were not statistically significantly altered in the presence of rhesus macaque-derived 90K-myc (Fig. 2A). Proteolytic cleavage of the glycoprotein precursor, however, was impaired by 90K proteins of all the nonhuman primate species, including the inactive rhesus macaque protein, by 25 to 39%, while Gag processing remained intact (Fig. 2B). Of note, levels of viral gp120 incorporation and of mature gp120 expression both correlated positively with the relative particle infectivity (Fig. 2C and D), and viral gp120 incorporation and gp160 processing correlated positively with each other (Fig. 2E). Similarly to gp160 processing, cell surface levels of HIV-1 gp120 were reduced by all 90K orthologs by 29 to 47% (Fig. 2F and G). Importantly, neither expression of human 90K nor expression of rhesus macaque 90K altered overall protein trafficking and secretion, as evidenced by the fact that extracellular concentrations of migration inhibition factor (MIF), macrophage colony-stimulating factor (M-CSF), and monocyte chemoattractant protein 1 (MCP-1) remained stable in the context of 90K expression (Fig. 2H), whereas they were effectively decreased by brefeldin A treatment, as assessed by Luminex-based multiplex assays (Fig. 2I). Taking these findings together, 90K's ability to interfere with glycoprotein precursor cleavage and to lower the pool of HIV-1 Env at the plasma membrane may be required, but not sufficient, for reduction of HIV-1 particle infectivity. Rather, specific impairment of incorporation of mature gp120 into nascent virions appears to be the primary mode of action of 90K.

### Specificity of anti-lentiviral activity of 90K.

**FIG 3:**
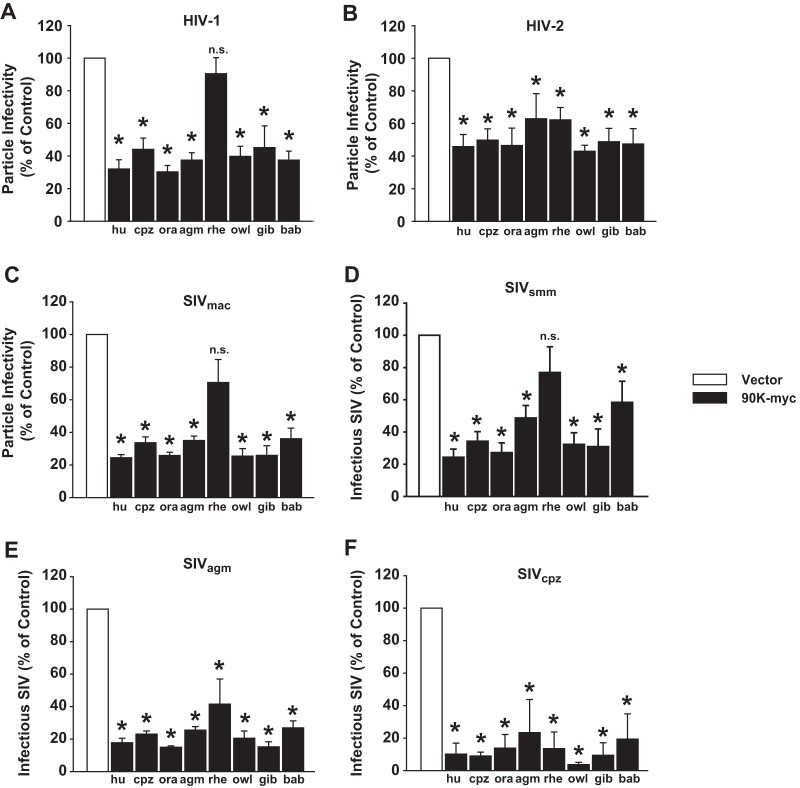
Specificity of antilentiviral activity of 90K. HEK293T cells were cotransfected with 1.3 μg HIV-1_NL4.3_ (A), HIV-2_7312A_ (B), SIV_mac_239 (C), SIV_smm_ (D), SIV_agm_ (E), or SIV_cpz_ (F) proviral DNA and 90K-myc-encoding expression plasmids of the indicated species. Asterisks indicate statistical significance (*P* < 0.05); n.s., not significant. (A to C) At 48 h posttransfection, the infectivities of cell-free HIV-1 (A), HIV-2 (B), and SIV_mac_239 (C) were calculated as infectivity per capsid antigen in the supernatant. Panel A is identical to Fig. 1B and is shown as a reference. (D to F) Due to the lack of availability of specific anti-CA antibodies, the mere relative infectivity is shown. Values obtained in the absence of 90K were set to 100%. Shown are mean values ± SEM from 4 to 7 independent experiments.

We next tested the ability of 90K-myc proteins to inhibit lentiviruses other than HIV-1. The particle infectivity of HIV-2 was reduced by all tested primate orthologs, including the rhesus macaque ortholog, albeit to milder extents than that of HIV-1 (Fig. 3A and B). Simian immunodeficiency virus from macaques (SIV_mac_) and SIV from sooty mangabey monkeys (SIV_smm_) were inhibited by all tested primate 90K proteins, with the exception of rhesus macaque-derived 90K, which only mildly reduced the infectivity of these SIVs to a level comparable to that for HIV-2, without, however, reaching statistical significance (Fig. 3C and D). SIV from African green monkeys (SIV_agm_) and SIV from chimpanzees (SIV_cpz_) were susceptible to inhibition by all tested 90K proteins, including the rhesus macaque-derived factor (Fig. 3E and F). However, due to the lack of availability of antibodies recognizing SIV_smm_, SIV_agm_, and SIV_cpz_ capsids, we cannot rule out an inhibitory effect of 90K on the release of these viruses, which potentially contributes to the small amount of infectivity released into the supernatant. These data indicate that inhibition by human and most nonhuman primate 90K proteins is not HIV-1 specific but is also directed against other lentiviruses. Among primate 90K proteins, rhesus macaque 90K is unique in its inability to diminish the particle infectivity of the tested HIV-1 strain and in its low potency against SIV_mac_ and SIV_smm_ strains, despite 89-to-97% amino acid identity with active primate 90K proteins.

### Toward a minimal antiviral 90K variant.

**FIG 4:**
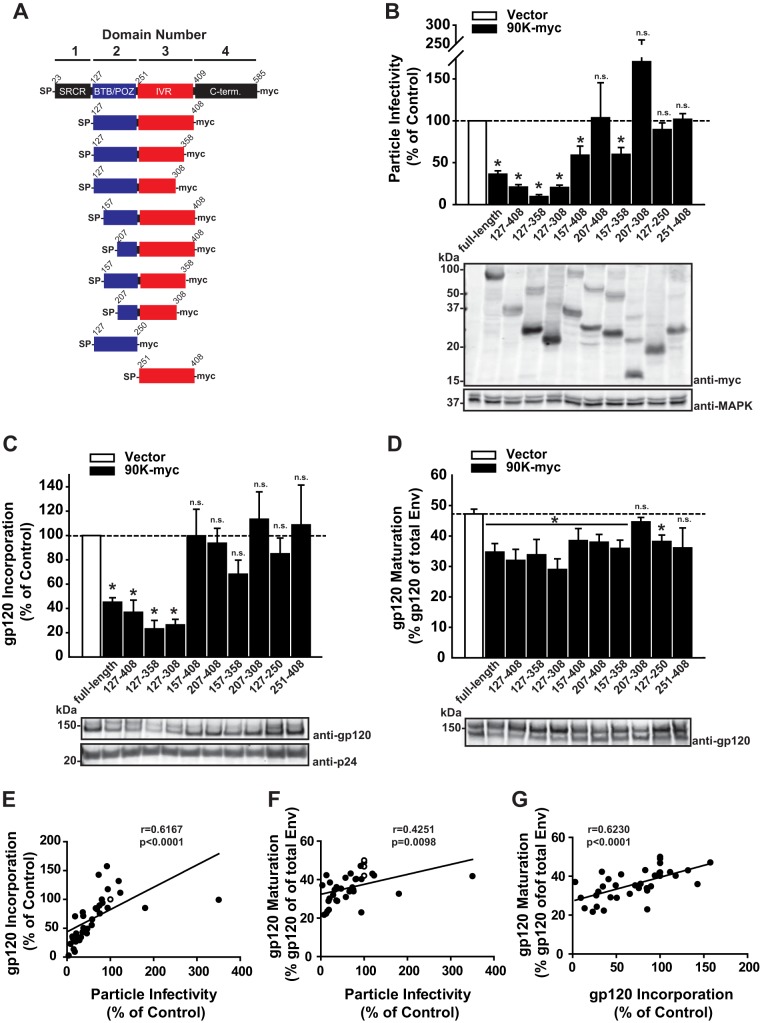
Toward a minimal antiviral 90K variant. (A) Scheme of the 90K protein domain organization and the analyzed truncated mutants. Numbers indicate amino acid positions. (B) HEK293T cells were cotransfected with HIV-1 proviral DNA and the indicated 90K constructs. At 48 h posttransfection, the infectivity of cell-free particles was measured as infectivity per nanogram of p24 capsid antigen in the supernatant. Values obtained in the absence of 90K were set to 100%. Shown are arithmetic means ± SEM from 3 to 8 independent experiments. Asterisks indicate statistical significance (*P* < 0.05); n.s., not significant. Lysates of the producer cells were immunoblotted with anti-myc and anti-MAPK antibodies. (C) Sucrose cushion-purified progeny virions were analyzed by immunoblotting, and band intensities were quantified by infrared-based imaging. gp120 incorporation, defined as the ratio of gp120 signal intensity to p24 signal intensity, was calculated from 3 to 6 independent experiments. Values obtained in the absence of 90K were set to 100%. (D) gp160 processing in cell lysates was determined by immunoblotting using the indicated antibodies. Band intensities were quantified by infrared-based imaging, and the percentage of mature gp120 from total Env protein is depicted. The bar diagrams show the arithmetic means ± SEM from 3 to 5 independent experiments. (E) Relative levels of gp120 viral incorporation and particle infectivity, obtained in the presence of the human 90K variants used, are plotted against each other. (F) Levels of mature gp120 per whole-Env expression in producer cell lysates and relative levels of particle infectivity are plotted against each other. (G) Relative levels of gp120 viral incorporation and levels of mature gp120 per whole-Env expression in producer cell lysates are plotted against each other. For these values, the Pearson correlation coefficient *r* and the corresponding *P* value are indicated. The open circles depict the values obtained in the absence of 90K expression.

We have shown previously that a shortened version of human 90K-myc, comprising solely domains 2 and 3 [hu 90K(127–408)], is at least as active as wild-type 90K-myc (9). Starting from this observation, we aimed at further narrowing down the minimal region required for antiviral activity. For this purpose, we generated a set of shortened variants of 90K-myc (Fig. 4A). Confirming our previous results (9), particle infectivity was slightly more efficiently reduced by 90K(127–408) than by wild-type 90K (Fig. 4B). Further C-terminal truncation [90K(127–358) and 90K(127–308)] did not curtail the antiviral activity. On the other hand, further stepwise truncation of the N terminus slightly decreased [90K(157–408)] or abrogated [90K(207–408)] antiviral activity and reversed the gain of antiviral activity introduced by C-terminal truncation [90K(157–358) and 90K(207–308)]. Finally, domains 2 and 3 expressed individually did not exhibit any antiviral activity (Fig. 4B), suggesting that a structural entity consisting of both domains is essential for full antiviral potential.

Expression of authentic 90K and the three highly active 90K versions [90K(127–408), 90K(127–358), and 90K(127–308)] resulted in secretion of virus particles displaying ≤80% reduced amounts of mature incorporated gp120. In contrast, expression of mildly active [90K(157–408), 90K(157–358)] and inactive [90K(207–408), 90K(207–308), 90K(127–250), and 90K(251–408)] 90K versions resulted in particles with normal gp120 Env (Fig. 4C). Proteolytic processing of the gp160 precursor was diminished by most shortened 90K versions, including some displaying mild or even absent antiviral activity (Fig. 4D). Finally, levels of incorporated gp120 and of mature gp120 expression both correlated positively with the relative particle infectivity (Fig. 4E and F), and levels of incorporated gp120 and of mature gp120 expression correlated positively with each other (Fig. 4G). These results reinforce the notions that the antiviral effect of 90K is mediated primarily via reduction of HIV-1 Env incorporation and that the observed interference with HIV-1 Env gp160 precursor processing may be required but is not sufficient.

### Genetic humanization renders a minimal version of rhesus macaque 90K antiviral.

**FIG 5:**
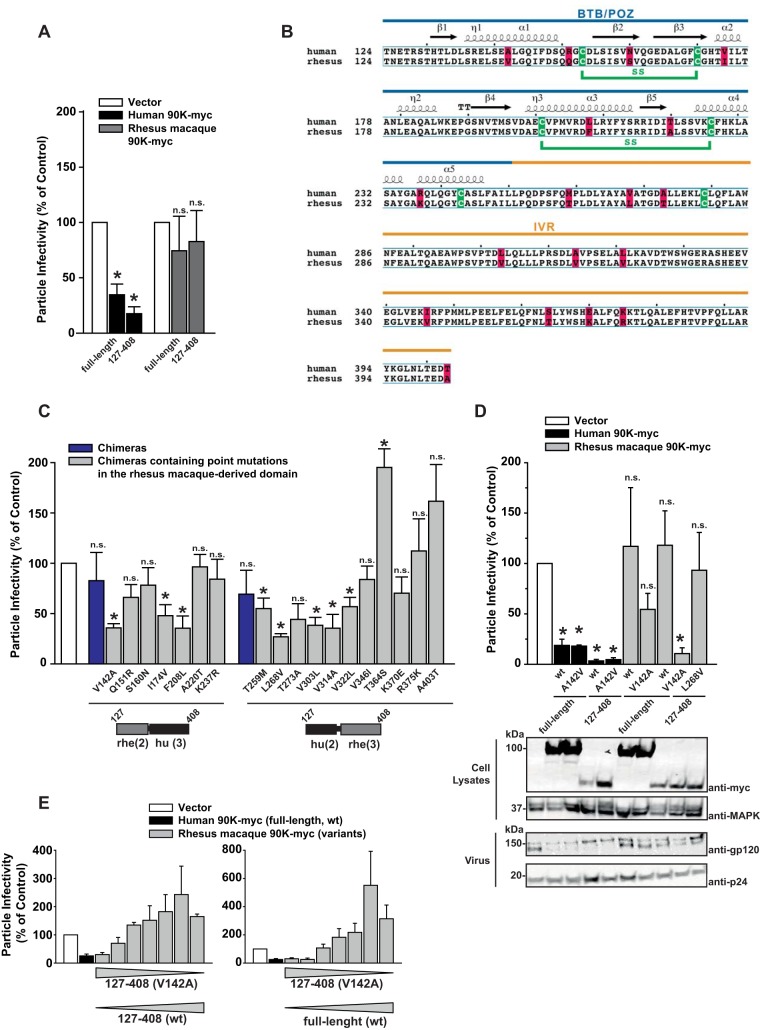
Genetic humanization renders a minimal version of rhesus macaque 90K antiviral. (A) HEK293T cells were cotransfected with HIV-1 proviral DNA and the indicated 90K constructs. At 48 h posttransfection, the infectivity of cell-free particles was measured as infectivity per nanogram of p24 capsid antigen in the supernatant. Values obtained in the absence of 90K were set to 100%. Shown are arithmetic means ± SEM from 3 to 8 independent experiments. Asterisks indicate statistical significance (*P* < 0.05); n.s., not significant. (B) The human and rhesus macaque BTB/POZ and IVR domains were aligned using Clustal Omega (41) and ESPript (42). Conserved secondary-structure elements, including α-helices, 3_10_-helices, β-strands, and β-turns taken from the human BTB domain structure, are shown above the alignment (α, η, β, and TT, respectively). Nonconserved residues are highlighted by a red background; a green background indicates conserved cysteines, and their disulfide connectivity is drawn in green below the alignment. (C) HEK293T cells were first cotransfected with HIV-1 proviral DNA and constructs encoding the indicated interspecies chimeras and then processed as for panel A. Shown are arithmetic means ± SEM from 3 independent experiments. (D) HEK293T cells were cotransfected with HIV-1 proviral DNA and the indicated constructs and were processed as for panel A. Shown are arithmetic means ± SEM from 3 independent experiments. (E) HEK293T cells were cotransfected with HIV-1 proviral DNA and combinations of plasmids encoding for short versions of wild-type rhesus macaque 90K-myc [127-408 (wt)] and of rhesus macaque 127-408 (V142A). Both 90K variants were titrated in the opposite direction, covering plasmid ratios of 0+4, 1+3, 1.5+2.5, 2+2, 2.5+1.5, 3+1, and 4+0. Transfected cells were processed as for panel A.

Based on interspecies comparison (Fig. 1) and analysis of minimal human 90K variants (Fig. 4), we embarked on a fine-mapping study with the goal of defining single amino acids that are crucial for 90K's antiviral activity. A minimal version of 90K from rhesus macaques, expressing solely domains 2 and 3 [rhe 90K(127–408)] was inactive, whereas its human counterpart showed strong antiviral activity (Fig. 5A). The two orthologs differ by 18 amino acids (aa) dispersed on both domains in this region (Fig. 5B). Chimeric constructs consisting of human domain 2 and rhesus macaque domain 3 or vice versa displayed no antiviral activity (Fig. 5C, blue bars). We then introduced individual, “humanizing” point mutations into the rhesus macaque portions of these chimeric constructs. Indeed, selected individual mutations in either rhesus macaque domain resulted in a gain of antiviral activity, with V^142^A and F^208^L in rhesus macaque domain 2 and L^268^V in rhesus macaque domain 3 representing the most influential exchanges in each construct, respectively (Fig. 5C). In order to probe whether selected amino acid exchanges are sufficient for rescuing the antiviral activity in the context of the authentic rhesus macaque protein, we transferred them individually into the full-length protein and into the short version of rhesus macaque 90K-myc [rhe 90K(127–408)] (Fig. 5D). Interestingly, introduction of V^142^A, but not L^268^V, into the short version of rhesus macaque 90K-myc resulted in a gain of antiviral activity that was in the same range as the short human version, and coincided with reduced gp120 incorporation (Fig. 5D). Although V^142^A introduction into the full-length rhesus macaque protein decreased the infectivity of virus particles 2-fold, these values did not reach statistical significance, pointing toward additional determinants in the scavenger receptor cysteine-rich (SRCR)-like domain and the C-terminal domain of the rhesus macaque factor that may negatively regulate antiviral activity. In addition, no loss of antiviral activity in either the full-length or the short version of human 90K was observed after the introduction of the reciprocal, “simianizing” A^142^V change (Fig. 5D), suggesting that other residues in the human context can compensate for V^142^. Titration of active and inactive rhesus macaque 90K constructs indicated that active variants can be outbalanced by inactive constructs (Fig. 5E).

### Crystal structure of the BTB/POZ domain of human and rhesus macaque 90K.

Given that a single amino acid exchange in the BTB/POZ domain of the short version of rhesus macaque 90K was sufficient to restore antiviral activity, we next set out to structurally characterize the environment of this residue within the short versions of the rhesus macaque and human 90K proteins. However, 90K has a tendency to oligomerize beyond homodimerization by forming ring-like structures of variable size (15), and this tendency was also observed for a 90K construct encompassing domains 2 and 3 (data not shown), rendering the structure analysis complicated. Since the individual BTB/POZ domains of both the human and rhesus macaque 90K proteins do not feature a similar oligomerization tendency, and given that a single amino acid exchange in the BTB/POZ domain of the short version of rhesus macaque 90K was sufficient to restore antiviral activity, we determined the structures of the individual BTB/POZ domains from the human and rhesus macaque 90K proteins. We expressed modified 90K BTB/POZ domains that contained three additional residues at the N terminus (aa 124 to 250; based on secondary structure predictions and comparisons with available structures of other BTB/POZ domains) in Drosophila melanogaster S2 cells and purified both proteins, which behaved as a single oligomeric species in solution, to homogeneity. We obtained crystals of the human protein that diffracted to 2.1 Å (crystallographic statistics are listed in Table 1) and crystals of the rhesus macaque protein diffracting to 3.3 Å. The structure of the human ortholog was determined by the multiwavelength anomalous dispersion (MAD) method as described in Materials and Methods. The human 90K BTB/POZ domain structure was subsequently used as a search model to determine the structure of the rhesus macaque ortholog by the molecular replacement method. The human and rhesus macaque structures contained 1 and 3 homodimers per asymmetric unit, respectively. For further analysis, the rhesus homodimer comprising all residues with the lowest mean B value after crystallographic refinement, indicating the highest degree of order in this part of the structure, was selected.

**TABLE 1:** Data collection, phasing, and refinement statistics

Parameter^*a*^	Value(s) for 90K-BTB^*b*^	
	Human	Rhesus macaque
Data collection statistics		
Space group	*P* 3_1_ 21	*I* 4_1_ 22
Cell dimensions		
*a*, *b*, *c* (Å)	70.34, 70.34, 105.51	181.76, 181.76, 173.52
α β, γ (°)	90.0, 90.0, 120.0	90.0, 90.0, 90.0
No. of molecules/AU	2	6
Resolution (Å)	50–2.08 (2.21–2.08)	50–3.3 (3.39–3.3)
*R*_meas_	0.12 (1.591)	0.141 (8.242)
*I/*σ (*I*)	14.46 (1.42)	21.70 (1.05)
*CC*_1/2_	0.99 (0.79)	1.00 (0.39)
Completeness (%)	99.2 (94.9)	100.0 (99.9)
Redundancy	18.6 (17.2)	56.9 (58.7)
Refinement statistics		
Resolution (Å)	20.77–2.08 (2.21–2.08)	49.17–3.3 (3.46–3.3)
No. of reflections	18,506	22,100
*R*_work_/*R*_free_	0.208/0.243	0.301/0.287
No. of atoms		
Protein	1,861	5,546
Solvent	41	0
*B* factors (Å^2^), protein	50.62	195.59
RMS deviations		
Bond length (Å)	0.010	0.007
Bond angle (°)	1.03	0.93
Ramachandran plot^*c*^ (%)		
Favored	99.2	96.1
Allowed	0.8	3.6
Outliers	0	0.3

a One crystal was used to collect each of the diffraction data sets used to determine the crystal structures. AU, asymmetric unit; *CC*_1/2_, Pearson correlation coefficient between two random half data sets.

b Values in parentheses are those for the highest-resolution shell.

c Ramachandran statistics were calculated with MolProbity.

**FIG 6:**
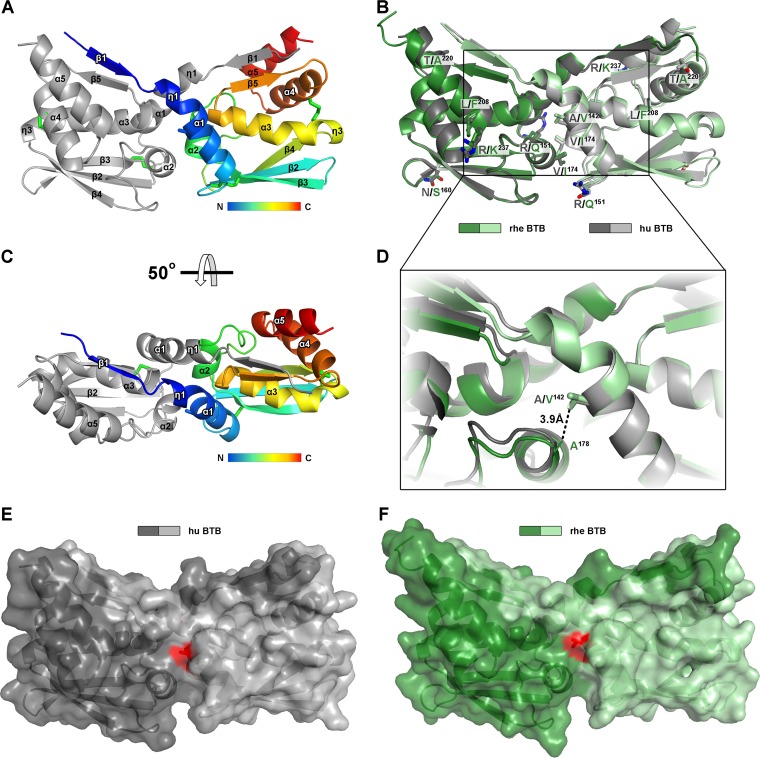
Crystal structure of human and rhesus macaque BTB. (A and C) Ribbon diagrams of the human BTB homodimer, with one subunit shown in gray and the other colored in gradations from the N to the C terminus (blue to red) according to the color scales in the bars underneath. Disulfide bonds are shown as green sticks. (B) Superposition of human BTB and rhesus macaque BTB, with the two subunits displayed in gray (human BTB) and green (rhesus macaque BTB). Side chains of residues that are divergent between the human and rhesus macaque 90K proteins are shown as sticks and are colored according to atom type (gray and green for carbon of human BTB and rhesus macaque BTB, respectively; red and blue for oxygen and nitrogen, respectively). (D) Closeup view of the homodimerization interface of superposed human BTB and rhesus macaque BTB. Side chains of residue 142, which provides a gain of antiviral activity in the context of the short version of rhesus macaque 90K-myc, and the conserved residue A^178^, which makes an additional hydrophobic contact with V^142^ (dashed line), are shown as sticks. (E and F) The molecular surfaces of the human (E) and rhesus macaque (F) 90K BTB domain are displayed in gray and green, respectively, as in panel B. The surface area corresponding to A^142^ (human BTB) or V^142^ (rhesus macaque BTB) is shown in red.

The experimental electron density maps of both BTB/POZ domains allowed us to trace the entire molecule comprising residues 129 to 250. The resulting atomic models revealed tightly intertwined homodimers with the canonical BTB/POZ homodimer fold, consisting of both α-helices and β-sheets (Fig. 6A and C). As expected from the sequence alignment, the two BTB/POZ domains are structurally conserved, with a DALI score of 22.6 and root mean square deviations (RMSD) of <0.6 Å for pairwise comparisons of 119 structurally equivalent Cα atoms. The seven residues that are divergent between the domains of the two species are distributed over the entire molecule (Fig. 5B and 6B).

Residue 142 (alanine in human 90K, valine in rhesus 90K), the mutation of which resulted in a gain of antiviral activity in the context of the short variant of rhesus macaque 90K, is located on helix α1 and participates in the homodimer interface. The buried interface areas of the hydrophobic V^142^ (rhesus) and A^142^ (human) residues in the respective BTB domain homodimer interfaces amount to 40.90 and 34.58 Å^2^ for the rhesus and human BTB domains, respectively, suggesting that a V^142^A mutation does not significantly modulate homodimer stability. In line with this observation, the calculated binding energies contributed by these two residues to homodimer formation (0.64 and 0.5 kcal/mol for V^142^ and A^142^, respectively) do not differ significantly, supporting the hypothesis that the differential antiviral activity of human and rhesus 90K proteins is not related to differential stability of the homodimer. In contrast, the accessible surface area of residue 142 within the homodimer (i.e., the area that is accessible at the surface of a dimerized BTB domain) differs by >15 Å^2^ (47.66 Å^2^ and 64.08 Å^2^ for the human and rhesus BTB domains, respectively), suggesting that the introduction of valine at this position results in a larger hydrophobic patch at the surface of the rhesus BTB domain that potentially stabilizes a direct interaction with either a different protein or another domain of 90K, thereby interfering with antiviral activity.

## DISCUSSION

Given the high degree of sequence conservation between 90K proteins from primate species, the overall preservation of the antiviral activity of 90K against lentiviruses was expected. However, as a notable exception, 90K protein from rhesus macaques failed to reduce HIV-1, SIV_mac_, or SIV_smm_ infectivity, despite sharing 89-to-97% amino acid identity with its primate counterparts. Interestingly, HIV-2, SIV_agm_, and SIV_cpz_ were sensitive to all primate 90K proteins, including the rhesus macaque-derived protein, arguing against a general misfolding or nonfunctionality of the latter. Although human 90K is antiviral against all R5 and X4 HIV-1 strains tested, reaching infectivity reduction ranges between 65 and 96% by heterologously expressed 90K and up to 91% by endogenous 90K (9), it will be interesting to analyze, in the future, whether different HIV-1 strains display different species specificity with regard to 90K-mediated inhibition, and whether this can be modulated by heterologous exchange of Env. Along this line, analysis of HIV-1–HIV-2 chimeric viruses may help to explain the overall inferior susceptibility of HIV-2 to 90K-mediated restriction, compared to HIV-1.

Analysis of active and inactive 90K proteins, either derived from different primate species or engineered by mutation, revealed that modest impairment of HIV-1 gp160 processing and reduction of cell surface pools of HIV-1 Env are conserved between all tested primate 90K proteins, including the inactive rhesus macaque protein. Furthermore, these features are preserved in most shortened versions of human 90K. We thus conclude that mere inhibition of gp160 processing and lowering of cell surface HIV-1 Env expression at observed efficiencies do not directly imply reduced infectivity of virions. In contrast, 90K-mediated reduction of the abundance of mature HIV-1 Env glycoproteins seems to be the essential parameter directly linked to antiviral activity. Importantly, protein trafficking and secretion remained intact in cells expressing human or rhesus 90K, excluding the possibility of a general influence of 90K proteins on the efficiency of these cellular processes. These data are in line with our earlier notion of unaltered surface CD4 protein expression (9) and fully maintained metabolic activity (9), as well as with the lack of modulation of HIV-1 p24 capsid secretion in HIV-1-producing, 90K-expressing cells. Although we favor the idea of a role of 90K in inhibiting gp120 incorporation, we cannot formally exclude the possibility that 90K induces, at least partially, instability and/or shedding of virus-associated Env.

As one possible conclusion, impairment of gp160 processing and reduction of cell surface HIV-1 Env amounts may be required, but not sufficient, for mediating the defect in gp120 incorporation and for exerting antiviral activity. Alternatively, the former features may represent a “bystander” effect of 90K that is uncoupled from their ability to impede viral incorporation of mature glycoproteins. From a broader perspective, these findings suggest that incorporation of HIV-1 glycoproteins may be efficient even under conditions in which only low numbers of mature Env glycoproteins are being synthesized. In contrast, reduction of viral incorporation of mature Env glycoproteins to levels below the reported amounts of 7 to 14 trimers of HIV-1_MN_ Env per virion (22, 23) appears to represent a vulnerable point of lentiviral replication and might represent a target for antiviral drug development in the future.

Interspecies comparison, paired with the analysis of minimal versions of 90K, led to the finding that “humanization” of the BTB/POZ domain at position 142 in the context of a minimal version of rhesus macaque 90K, consisting solely of domains 2 and 3 of the authentic factor [rhe 90K(127–408)], was sufficient to restore antiviral activity. While we identified, in total, three amino acid exchanges in the BTB/POZ domain that conferred antiviral activity on the previously inactive rhesus macaque-human chimera, V^142^A represented the most potent exchange. Although this is a conservative amino acid exchange, documented examples of V-to-A polymorphisms illustrate profound biological relevance. These include the V^226^A exchange in the Chikungunya virus (CHIKV) E1 protein, conferring the ability of the virus to expand its vector usage (24); the acquisition of resistance by HIV-1 Gag to antiviral compounds through an A-to-V change in the CA/SP1 cleavage site of Gag (25); and the abolishment of signaling by the ATP-binding cassette transporter A1 through the introduction of A^937^V (26). The structures of both BTB/POZ domains showed that residue 142 is located at the edge of the homodimer interface, but the larger hydrophobic valine side chain results in a larger hydrophobic patch at the surface of the homodimer. The gain of function induced by V^142^A in rhe 90K(127–408) could originate either from a direct effect of the mutation (e.g., a direct interaction) or from an indirect effect (e.g., modification of the 90K oligomeric nature affecting protein-protein interactions). It remains elusive whether the observed effect is due to interaction with a protein partner or with a different 90K domain. Although previous attempts failed to demonstrate an interaction of HIV-1 Env and human 90K in coimmunoprecipitation experiments (9), we cannot rule out the possibility of a weak or transient interaction between Env and 90K, which may contribute to, or even be crucial for, 90K's antiviral activity. In the future, it will be necessary to identify potential species-specific cellular or viral factors which may interact with 90K in a manner that modulates antiviral activity. A translational perspective may consist of reinforcing 90K's antiviral potential in order to render it more effective, e.g., by specifically increasing its expression levels or by designing cell-permeant small molecules that stabilize a cellular interaction that is essential for the antiviral mode of action. Future studies should also elucidate whether HIV-1 can evolve to full 90K resistance after long-term passage in 90K-expressing cells and should determine the genomic location of the viral escape mutations, allowing the design of specific antiviral strategies.

V^142^A was not sufficient to entirely restore antiviral activity to the full 90K protein from rhesus macaques, suggesting that yet-to-be-identified determinants within the N-terminal SRCR domain and the poorly characterized C-terminal domain negatively regulate the antiviral capacity of rhesus macaque 90K. Adding another layer of complexity, the antiviral 90K proteins from African green monkeys and baboons share V^142^ with the inactive 90K protein from rhesus macaques (data not shown). In addition, no loss of antiviral activity was observed for either full-length human 90K or hu 90K(127–408) after introduction of the reciprocal, “simianizing” A^142^V exchange, suggesting that other residues in the human protein context can compensate for V^142^. Taken together, our results suggest that a particular quaternary arrangement of 90K domains 2 and 3 is required for antiviral activity. The interface stabilizing this arrangement likely involves residue 142 within the BTB/POZ domain, and in the rhesus context, a valine at this position interferes with this functional quaternary arrangement. Of note, the antiviral activities of 90K proteins from African green monkeys and baboons, as well as that of “simianized” A^142^V human 90K, indicate that this interference can be overcome in the context of other 90K orthologs, likely due to other residues involved in the quaternary interface. Future studies to elucidate the molecular mechanism of species-specific antiviral activity should therefore aim for a structural characterization of full-length 90K, or at least a 90K construct encompassing domains 2 and 3 at atomic resolution. However, 90K has the tendency to oligomerize beyond homodimerization by forming ring-like structures of variable size (15), rendering structure analysis complicated. This tendency has also been observed for a 90K construct encompassing domains 2 and 3 (data not shown).

Finally, while our study specifically investigated the species specificity of 90K regarding the direct interference with HIV-1 particle infectivity, further work is required to identify or exclude potential species-specific differences regarding interferon (IFN)-mediated regulation of 90K expression and abilities to induce antiviral and inflammatory cytokines.

In conclusion, we established the species specificity of the antiviral protein 90K, and species-specific comparison enabled us to obtain important insight into its antiviral mechanism. Further studies are required in order to characterize 90K's detailed mode of action and to elucidate potential evasion strategies that may emerge in virus strains that are forced to propagate in 90K-expressing cells.

## MATERIALS AND METHODS

### Cells.

HEK293T cells (obtained from the American Type Culture Collection [ATCC]) and TZM-bl cells (obtained from the NIH AIDS Reagent Program) were cultured as recommended. HEK293T cells were transfected by calcium phosphate DNA precipitation (Clontech) or with polyethylenimine (PEI) (Polyplus, Illkirch, France) as a transfection reagent. Brefeldin A was purchased from BioLegend and was used at a final concentration of 5 μg/ml.

### Plasmids.

pcDNA6 and pcDNA6.90K-myc plasmids were kindly provided by Ji Hee Lee, South Korea (27). cDNAs encoding 90K-myc orthologs (cpz, chimpanzee [Pan troglodytes]; ora, orangutan [Pongo pygmaeus]; agm, African green monkey [Cercopithecus aethiops tantalus]; rhe, rhesus macaque [Macaca mulatta]; owl, owl monkey [Aotus trivirgatus]; gib, gibbon, [Hylobates lar]; bab, baboon [Papio hamadryas]) were amplified by PCR and cloned into the pcDNA6/myc-His vector backbone using BamHI*/*XbaI sites. The shortened variants of 90K-myc all contained the authentic signal peptide and were generated by insertion into the pcDNA6/myc-His backbone using BamHI and XbaI or HindIII and XbaI. Chimeras were generated by splicing by overhang extension PCR (SOE-PCR) and were inserted in the pcDNA6/myc-His backbone using BamHI/XbaI sites. For SOE-PCR, first, domains of each species were amplified using primers with overlaps for the proximate domains of the various species, respectively. In a following PCR, products with overlaps were amplified to one chimeric product. Single point mutations were introduced by site-directed mutagenesis (Stratagene). The proviral HIV-1_NL4.3_ DNA was obtained from Oliver Keppler. The expression plasmid for proviral HIV-2_7312A_ was provided by Beatrice Hahn.

### Lentiviral particle infectivity assay.

The infectivity of lentiviral particles was assessed by applying a standardized TZM-bl-based firefly luciferase assay or beta-galactosidase assay. Readouts were obtained luminometrically (RLU, relative light units). For the calculation of HIV-1 and HIV-2 particle infectivity, luminometric counts were divided by the content of p24, which was quantified by an anti-HIV-1 p24 enzyme-linked immunosorbent assay (ELISA). For the calculation of SIV_mac_ particle infectivity, luminometric counts were divided by the content of p27, which was quantified by anti-SIV_mac_ p27 ELISA. Luminometric activity was analyzed with a Centro LB 960 Microplate luminometer and Ascent 2.0 software.

### Anti-p24 and anti-p27 antigen ELISA.

For quantification of HIV-1 p24 capsid antigen in the culture supernatant, a homemade sandwich ELISA was used (28). A commercial ELISA (ABL, Inc.) was used for detection of SIV_mac_239 p27 capsid antigen.

### Antibodies.

The following antibodies/antisera were used for immunoblotting: mouse anti-myc (provided by Jens von Einem), goat anti-myc (Novus Biologicals), rabbit anti-mitogen-activated protein kinase (anti-MAPK) (Santa Cruz), mouse anti-HIV-1 p24 (Exbio), and rabbit anti-HIV-1 gp120 (provided by Valerie Bosch). For HIV-1 Env staining on nonpermeabilized cells, experiments were performed with anti-gp120 antibody 2G12 or 10-1074 (obtained from the NIH AIDS Reagent Program).

### Immunoblotting.

Cells were lysed with M-PER Mammalian protein extraction reagent (Pierce), and virions were prepared from supernatants by ultracentrifugation through a 20% (wt/vol) sucrose cushion. Proteins were run on a 12.5% or 7.5% SDS-PAGE gel and were transferred to nitrocellulose membranes. Blocked membranes were incubated with primary antibodies. Secondary antibodies conjugated to Alexa Fluor 680/800 fluorescent dyes were used for detection by the Odyssey Infrared Imaging System (Li-Cor Biosciences) with quantification by Odyssey software.

### HIV-1 Env surface staining.

Upon transient cotransfection with proviral plasmids encoding green fluorescent protein (GFP) and 90K-myc-encoding constructs, HEK293T cells were harvested after 48 h. Cells were stained with the primary anti-gp120 antibody (2G12) followed by secondary staining with the Alexa Fluor 647-conjugated goat anti-human IgG (Invitrogen) (9, 29). After the surface HIV-1 Env staining, cells were fixed with paraformaldehyde (PFA) and analyzed for surface HIV-1 Env levels. Due to the disproportional expression of Env inside the cell (high) as opposed to on the cell surface (low) in provirally transfected HEK293T cells (4, 9), we restricted the analysis to gate R3, which contains cells driving sufficiently high viral gene expression to yield detectable cell surface Env, and normalized these signals to the respective R2 gate. Flow cytometry analysis was performed using FACSCalibur with BD CellQuest Pro 4.0.2 software (BD Pharmingen) and FlowJo V10 software (FlowJo).

### Expression and purification of the 90K BTB domains.

The cDNA corresponding to the BTB domains of human and rhesus macaque 90K proteins, comprising residues 124 to 250, was cloned into a modified Drosophila S2 expression vector described previously, and transfection was performed as reported earlier (30). For large-scale production, cells were induced with 4 mM CdCl_2_ at a density of approximately 7 × 10^6^ cells/ml for 6 days and were pelleted, and the soluble ectodomain was purified by affinity chromatography from the supernatant using a StrepTactin XT Superflow column followed by size exclusion chromatography (SEC) using a Superdex 200 column in 10 mM Tris (pH 8.0), 100 mM NaCl. For crystallization purposes, the affinity tag was removed by enterokinase digestion (EKMax; Invitrogen, San Diego, CA, USA) according to the manufacturer's instructions, followed by removal of uncleaved protein using Strep-Tactin affinity chromatography and SEC. In addition, human BTB was enzymatically deglycosylated using endoglycosidase D (New England Biolabs, Frankfurt, Germany) according to the manufacturer's instructions, followed by removal of the glycosidase and the cleaved sugar chains by SEC. Pure protein was concentrated to approximately 6 mg/ml.

### Crystallization and structure determination.

Crystals of BTB were grown at 293K using the hanging-drop vapor diffusion method in drops containing 1 μl purified protein (6 mg/ml in 10 mM Tris [pH 8.0], 100 mM NaCl) mixed with 1 μl reservoir solution containing 29% polyethylene glycol 4000 (PEG 4000), 0.2 M MgCl_2_, 0.1 M Tris (pH 8.5), and 10% sucrose (for rhesus macaque BTB) or 9% PEG 8000, 0.2 M zinc acetate, and 0.1 M imidazole (pH 6.8) (for human BTB). Diffraction quality rod-like crystals appeared after 4 to 5 days and were flash-frozen either in mother liquor (for rhesus macaque BTB) or in mother liquor containing 25% ethylene glycol (for human BTB). Data collection was carried out at PETRA III (DESY; P14) and the Synchrotron Soleil (Proxima2). Data were processed, scaled, and reduced with XDS (31), Pointless (32), and programs from the CCP4 suite (33). A multiwavelength anomalous dispersion (MAD) data set was collected from two independent volumes of a single human BTB crystal around the K-edge of zinc, and the structure was determined using the Phenix AutoSol Wizard (34). The structure of rhesus macaque BTB was determined by the molecular replacement method using Phaser (35) with the human BTB structure as the search model. Model building was performed using Coot (36), and refinement was done using AutoBuster (37) with repeated validation using MolProbity (38). Analyses of the relevant interfaces were performed using the jsPISA server (39).

### Data presentation and statistical analysis.

Unless stated otherwise, bars show the arithmetic means of results from the indicated number of repetitions. Error bars indicate standard errors of the means (SEM) from the indicated number of individual experiments. Statistical significance was calculated by performing a two-tailed Student *t* test using Excel. Pearson′s correlation analyses were performed with GraphPad Prism.

### Accession number(s).

The atomic coordinates and structure factors for two structures have been deposited in the Protein Data Bank (http://www.pdb.org/) under accession numbers 6GFB (human BTB) and 6GFC (rhesus macaque BTB).
